# Psychiatry, not mental health

**DOI:** 10.1192/bjb.2018.2

**Published:** 2018-02

**Authors:** Richard Braithwaite

**Affiliations:** Consultant Psychiatrist, Isle of Wight NHS Trust; email: richard.braithwaite@iow.nhs.uk

Timms begins his article on solid ground, highlighting the serious problem of psychiatric jargon.^1^ Sadly, it descends into a light-hearted list, focused on the gulf between manager and clinician in the British health service. Most importantly, he omits four gravely misused jargon terms prevalent in psychiatry.

First, the noun ‘depression’ and its adjective ‘depressed’. The noun has half a dozen dictionary definitions unrelated to medicine^2^ and, as a word on its own, it is not a recognised diagnosis in either psychiatric classification.^3^^,^^4^ Yet it is used by the lay public, patients and healthcare professionals alike to refer to a medical diagnosis, a single symptom or a normal emotion. It is invariably unclear which meaning is intended by the speaker. This confusion contributes to massive over-prescription of an unfortunately named class of drugs;^5^ anecdotally, it is not uncommon even for senior psychiatrists to justify antidepressant treatment, having cast aside clear diagnostic criteria,^3^^,^^4^ with comments such as: ‘Well, there's definitely a bit of depression there’ or ‘She says she feels depressed’.

In a similar vein, ‘paranoia’ and ‘paranoid’ are often used by clinicians in their lay meaning of ‘intense suspicion’,^2^ when the true psychiatric definition is ‘delusional’;^6^ such ideation might involve purely grandiose or somatic themes. Despite this, one often sees ‘paranoid’ and ‘persecutory’ used synonymously. The subjective complaint of ‘paranoia’ is common in patients with neurotic presentations and personality disorders, and its inappropriate use in case notes without careful use of inverted commas, to signify a verbatim quote, risks inappropriate labelling of patients as psychotic and overtreatment with antipsychotics.

Next, the term ‘psychosis’ is increasingly used as a diagnosis – as if it were a singular disease for which specific treatments were indicated^7^ – rather than the syndrome that it is. It can occur in organic, substance-induced or affective disorders, yet I contend that ‘psychosis’ is often used, lazily, as a euphemism for schizophrenia, by psychiatrists either ignorant of established diagnostic criteria^3^^,^^4^ or wary of stigmatising their patients (as if one would happily tell guests at a dinner party that one was ‘psychotic’).

Most concerning, though, is the jargon that Timms includes in his own title: ‘mental health’.^1^ The assumption that ‘mental health’ and the oxymoronic ‘mental health disorder’ are synonymous with psychiatry and its diseases is quite erroneous. Psychiatry, as practised by psychiatric nurses and psychiatrists, was once charged with the management of patients with psychiatric diseases. But our colleagues are now mental health nurses and our departments mental health services. Far from relating to recognised diseases, the doublespeak ‘mental health’ has become synonymous with a vague and unattainable concept of complete emotional well-being. Consequently, an increasing fraction of our population, even a majority according to some reports,^8^ young and old, are reported to have ‘mental health problems’. The jargon underlying this explosion has set us and our entire healthcare system up to fail, through unrealistic public expectations and ever unmet need.

Let us be psychiatrists and psychiatric nurses once more; let us work in psychiatric services. Let us diagnose schizophrenia and depressive episodes using recognised criteria and be judicious in our use of potentially hazardous and costly treatments; most of all, let us avoid terms steeped in ambiguity.
